# Neck management in metastatic cutaneous squamous cell carcinoma of the head and neck

**DOI:** 10.3389/fonc.2024.1344115

**Published:** 2024-02-29

**Authors:** Ning Xu, Qiang Sun

**Affiliations:** ^1^ Special Clinic, Henan Provincial Stomatological Hospital, The First Affiliated Hospital of Zhengzhou University, Zhengzhou, China; ^2^ Department of Oral and Maxillofacial Surgery, The First Affiliated Hospital of Zhengzhou University, Zhengzhou, China

**Keywords:** head and neck cutaneous squamous cell carcinoma, elective neck dissection, elective neck irradiation, parotid lymph node, observation

## Abstract

**Objective:**

Optimal neck management remains unclear in head and neck cutaneous squamous cell carcinoma (HNcSCC) with parotid metastasis. Our goal was to compare the impact of different cervical treatments on HNcSCC with parotid metastasis.

**Methods:**

Patients were retrospectively included. The primary outcome variables were regional control (RC) and disease-specific survival (DSS). The impacts of observation, elective neck irradiation (ENI), and elective neck dissection (END) were analyzed using the Cox model and presented as hazard ratios (HRs) and 95% confidence intervals (CIs).

**Results:**

In total, 268 patients were enrolled. In the Cox model for RC, compared with ENI, observation was associated with a significantly higher risk of regional recurrence (*p* = 0.001, HR = 2.50, 95%CI = 1.45–4.30). However, END showed a comparable influence on regional recurrence (*p* = 0.246, HR = 0.70, 95%CI = 0.38–1.28). In the Cox model for DSS, END demonstrated a similar HR of 0.62 (95%CI = 0.30–1.26) compared to ENI (*p* = 0.184). However, patients who underwent observation were associated with an additional nearly twofold risk of cancer-related mortality (HR = 2.85, 95%CI = 1.55–5.23). Subgroup analysis showed that ENI predicted comparable RC (*p* = 0.389) and DSS (*p* = 0.346) in patients with one or two metastatic parotid lymph nodes, but worse RC (*p* = 0.007) and DSS (*p* = 0.024) in patients with more than three positive lymph nodes.

**Conclusion:**

In HNcSCC with parotid metastasis, elective treatment of neck lymph nodes with END or ENI should always be performed.

## Introduction

Basal cell carcinoma is the most frequently encountered skin malignancy, and it generally carries a favorable prognosis as it exhibits an extremely low incidence of lymph node (LN) metastasis (1). Despite being less common than basal cell carcinoma, cutaneous squamous cell carcinoma of the head and neck (HNcSCC) is associated with a significant number of deaths related to skin cancer once there is LN metastasis (2). The parotid LN is the first drainage site for most HNcSCCs, which consists of intraparotid and periparotid LNs (3). Occult neck metastasis is one of the most important prognostic factors and is more common when there is parotid LN metastasis (P+), lymphovascular invasion (LVI), and perineural invasion (PNI) (4–7). It is widely accepted that neck dissection is required when a cN+ status occurs in P+ HNcSCC, but there remains controversy regarding neck management for a cN0 neck.

Both observation and elective neck treatment are viable options for patients with a cN0 neck. Decision analysis has described that an observation approach is justified in patients with a probability of occult metastases of <19% (8). A recent report has described that elective neck dissection (END) is not related to better survival in HNcSCC even when there is the presence of known histologic risk factors for lymphogenic spread due to their low positive predictive value (9). Observation could achieve a non-inferior prognosis to END (10), but some literature also suggests that END is needed for all patients with locally advanced HNcSCC (11). These findings reveal that there is still much unknown about the role of elective neck treatment in the prognosis of P+ HNcSCC.

Therefore, this study aimed to compare the impact of different neck management strategies on regional control (RC) and disease-specific survival (DSS) in P+ HNcSCC.

## Methods

### Ethical considerations

This study was approved by the First Affiliated Hospital of Zhengzhou University Institutional Research Committee (no. HNCR-20230150), and written informed consent for medical research was obtained from all patients prior to initial treatment. All methods were performed in accordance with relevant guidelines and regulations.

### Patient selection

From January 2000 to December 2022, patients with surgically treated primary HNcSCC were retrospectively reviewed. The inclusion criteria were as follows: there was pathologically parotid LN metastasis and a cN0 status was confirmed based on the eighth AJCC N stage. Patients without any follow-up were excluded. Data on demography, pathology, treatment, and follow-up were extracted and analyzed.

### Study variable

All pathological sections were reviewed by at least two head-and-neck pathologists. The tumor stage was determined according to the eighth edition of the AJCC system. A cN0 neck was determined using ultrasound and CT scans. LVI was defined as the presence of malignant cells within lymphatic or blood vessels. PNI was defined as the infiltration or spread of malignant cells along or within the nerves. Extranodal extension (ENE) was defined as the presence of cancer cells outside the LN capsule in the parotid LN (12). A positive margin was determined when there was evidence of residual tumor tissue or cells based on both frozen section analysis and postoperative pathology.

The primary outcome variables were the 5-year RC and DSS. Time to RC was calculated from the date of surgery to the date of first regional recurrence or last follow-up, while time to DSS was calculated from the date of surgery to the date of cancer-caused death or last follow-up.

### Treatment principle

In our department, primary tumor and parotid metastasis receive radical excision and superficial or total parotidectomy. We attempt to obtain a negative margin in every patient during the operation evaluated by frozen section, but if this could not be achieved, adjuvant therapy is performed.

Neck management in cN0 P+ HNcSCC comprised observation, END, and elective neck irradiation (ENI). The choice of which method was performed was usually based on the surgeon’s experience and the opinions of the patient’s family.

The presence of PNI, LVI, LN metastasis, positive margin, and ENE served as indications for adjuvant radiotherapy. After the operation, all patients underwent radiotherapy for the parotid area *via* mixed electrons or intensity-modulated radiotherapy; the field usually included partial levels I/II. In the ENI group, the field included at least I–III and Va, and the total dose was approximately 50–60 Gy. In the END group, patients with occult disease also received neck radiotherapy; the field included levels I–V, and radiotherapy of 60–70 Gy was delivered. The contralateral neck was not irradiated in all patients.

## Statistical analysis

The chi-square test was used to assess differences in the clinicopathological variables among the observation, END, and ENI groups. The impact of different neck managements on RC and DSS was evaluated *via* univariate and Cox models and presented as hazard ratios (HRs) with 95% confidence intervals (CIs). All statistical analyses were performed using R 3.4.4. A *p* < 0.05 was considered as significant.

## Results

### Baseline data

A total of 268 patients were included in our study. Of these individuals, 207 (77.2%) were men and 61 (22.8%) were women, with a mean age of 65 ± 15 years. Immunosuppression was noted in 17 (6.3%) patients secondary to bone marrow (*n* = 10) and solid organ (*n* = 7) transplantation. The primary site of the tumor was located in the ear or around it in 115 (42.9%) patients, the temporal region in 69 (25.7%) patients, the forehead in 53 (17.8%) patients, and other sites in 31 (11.6%) patients. The pathological tumor stage was T1/T2 in 201 (75.0%) patients and was T3/T4 in 67 (25.0%) patients. Tumors were well differentiated, moderately differentiated, and poorly differentiated in 92 (34.3%), 128 (47.8%), and 48 (19.4%) patients, respectively. PNI and LVI were detected in 31.0% and 29.1% of the patients, respectively. Positive margins were identified in 24 (9.0%) patients.

**Figure 1:**
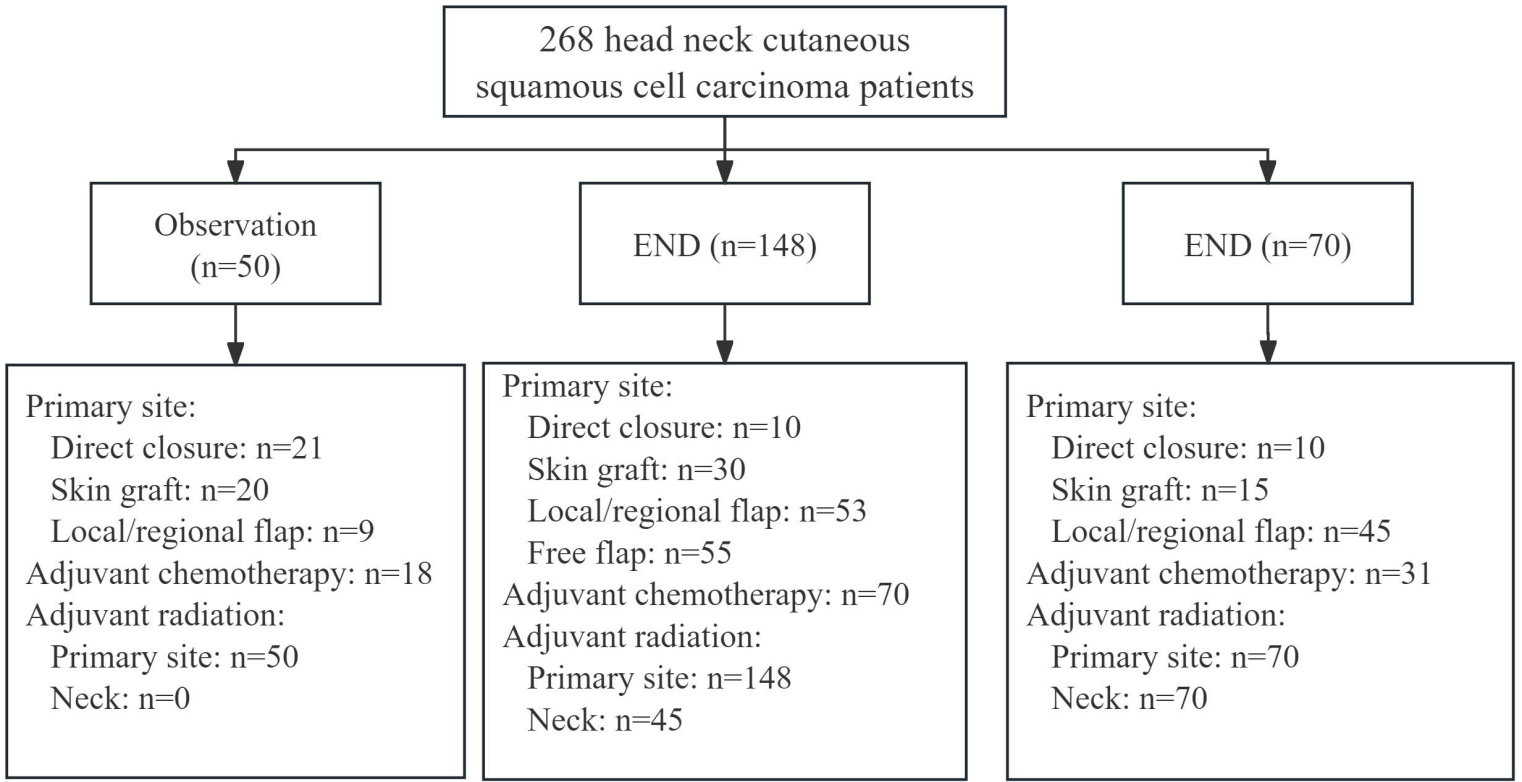
Flowchart of the three groups. HNcSCC, head neck cutaneous squamous cell carcinoma; END, elective neck dissection; ENI, elective neck irradiation.

Primary sites were treated by direct closure in 41 patients, skin graft in 65 patients, local or regional flap in 107 patients, and free flap in 55 patients. Superficial and total parotidectomy were performed in 80 (29.9%) and 188 (70.1%) patients, respectively. The deep lobe was involved in 68 (25.4%) patients. The number of metastatic parotid LNs was one in 123 patients, two in 79 patients, three in 36 patients, four in 15 patients, five in 10 patients, and six or more in 5 patients. The mean number of positive parotid LNs was 2. ENE of the parotid LN occurred in 110 (41.0%) patients. In the END group, occult metastasis was observed in 45 (30.4%) patients, all of whom subsequently underwent neck radiotherapy (**Figure 1**).

The three groups showed similar distributions of the clinicopathological factors (all *p* > 0.05), except for ENE, margin status, and parotidectomy. Patients treated with END tended to have a positive margin (*p* = 0.037) and ENE (*p* = 0.046) and to undergo total parotidectomy (*p* < 0.001) (**Table 1**).

**Table 1:** Comparison of the clinicopathological variables among observation, elective neck dissection (END), and elective neck irradiation.

Variable	Observation (*n* = 50)	END (*n* = 148)	ENI (*n* = 70)	*p*-value
Age (years)				
<65	24 (48.0%)	80 (54.1%)	35 (50.0%)	0.711
≥65	26 (52.0%)	68 (45.9%)	35 (50.0%)	
Gender				
Men	38 (76.0%)	117 (79.1%)	52 (74.3%)	0.716
Women	12 (24.0%)	31 (20.9%)	18 (25.7%)	
Immunosuppression	3 (6.0%)	10 (6.8%)	4 (5.7%)	1.000
Primary site				
Ear	15 (30.0%)	70 (47.3%)	30 (42.9%)	0.397
Temporal	13 (26.0%)	37 (25.0%)	19 (27.1%)	
Forehead	13 (26.0%)	26 (17.6%)	14 (20.0%)	
Others	9 (18.0%)	15 (10.1%)	7 (10.0%)	
Tumor stage				
T1 + T2	39 (78.0%)	108 (73.0%)	54 (77.1%)	0.692
T3 + T4	11 (22.0%)	40 (27.0%)	16 (22.9%)	
Differentiation				
Well	20 (40.0%)	50 (33.8%)	22 (31.4%)	0.072
Intermediate	17 (34.0%)	79 (53.4%)	32 (45.7%)	
Poor	13 (26.0%)	19 (12.8%)	16 (22.9%)	
PNI	14 (28.0%)	47 (31.8%)	22 (31.4%)	0.860
LVI	15 (30.0%)	43 (29.1%)	20 (28.6%)	0.985
Positive margin	0 (0.0%)	15 (10.1%)	9 (12.9%)	0.037
Parotid metastasis				
1–2	39 (78.0%)	110 (74.3%)	53 (75.7%)	0.870
3+	11 (22.0%)	38 (25.7%)	17 (24.3%)	
ENE	13 (26.0%)	68 (45.9%)	29 (41.4%)	0.046
Adjuvant chemotherapy	18 (36.0%)	70 (47.2%)	31 (44.3%)	0.380
Deep lobe involved	10 (20.0%)	38 (25.7%)	20 (28.6%)	0.563
Parotidectomy				
Superficial	37 (74.0%)	39 (26.4%)	35 (50.0%)	<0.001
Total	13 (26.0%)	109 (73.6%)	35 (50.0%)	
Occult metastasis	–	45 (30.4%)	–	

PNI, perineural invasion; LVI, lymphovascular invasion; ENE, extranodal extension.

### Univariate analysis

After follow-up with a mean time of 3.3 ± 1.4 years, a total of 10 local recurrences, 86 regional recurrences, and 66 cancer-caused deaths occurred. There were five local and 34 regional recurrences in the observation group, three local and 32 regional recurrences in the END group, and two local and 20 regional recurrences in the ENI group. The median regional recurrence times for the observation, ENI, and END groups were 1.9 (range = 0.2–4.5), 1.7 (range = 0.3–4.3), and 1.9 years (range = 0.5–3.7 years). The mean DSS times for the observation, ENI, and END groups were 3.5 ± 0.2, 4.4 ± 0.2, and 4.6 ± 0.1 years.

Factors of immunosuppression and margin status, tumor stage, number of positive parotid LNs, ENE, deep lobe involvement, and neck management were associated with RC and DSS (all *p* < 0.05; **Supplementary Figure S1**). Patients with PNI (*p* = 0.092) or poor differentiation (*p* = 0.090) tended to have worse DSS. Other variables did not impact RC or DSS (all *p* > 0.05) (**Table 2**).

**Table 2:** Univariate analysis of the predictors for regional control (RC) and disease-specific survival (DSS).

Variable	RC	DSS
Age (≥65 *vs*. <65)	0.573	0.422
Gender (men *vs*. women)	0.413	0.776
Immunosuppression (yes *vs*. no)	<0.001	<0.001
Primary site (ear *vs*. temporal *vs*. forehead *vs*. others)	0.107	0.233
Tumor stage (T3 + T4 *vs*. T1 + T2)	<0.001	<0.001
Differentiation (poor *vs*. intermediate *vs*. well)	0.405	0.090
PNI (yes *vs*. no)	0.100	0.092
LVI (yes *vs*. no)	0.392	0.115
Positive margin	<0.001	<0.001
No. of metastatic parotid lymph nodes (3+ *vs*. 1–2)	<0.001	0.036
Adjuvant chemotherapy	0.127	0.256
ENE	<0.001	<0.001
Deep lobe involvement	<0.001	<0.001
Parotidectomy (total *vs*. superficial)	0.456	0.287
Neck management (END *vs*. observation *vs*. ENI)	<0.001	<0.001

PNI, perineural invasion; LVI, lymphovascular invasion; ENE, extranodal extension; END, elective neck dissection; ENI, elective neck irradiation.

### Multivariate analysis

In the Cox model for RC, compared with ENI, observation was associated with a significantly higher risk of regional recurrence (*p* = 0.001, HR = 2.50, 95%CI = 1.45–4.30). However, END showed a comparable influence on regional recurrence (*p* = 0.246, HR = 0.70, 95%CI = 0.38–1.28). Other independent variables included immunosuppression (*p* = 0.002, HR = 5.11, 95%CI = 1.81–14.45), T3/T4 stage (*p* = 0.008, HR = 5.08, 95%CI = 1.54–16.79), positive margin (*p* < 0.001, HR = 9.93, 95%CI = 3.55–27.79), and three or more positive parotid LNs (*p* < 0.001, HR = 7.43, 95%CI = 3.32–16.62). ENE, or deep lobe involvement, did not influence RC (**Table 3**).

**Table 3:** Multivariate analysis of the predictors for regional control (RC).

Variable	RC	
	*p*-value	HR (95%CI)
Immunosuppression (yes *vs*. no)	0.002	5.11 (1.81–14.45)
Tumor stage (T3 + T4 *vs*. T1 + T2)	0.008	5.08 (1.54–16.79)
Positive margin	<0.001	9.93 (3.55–27.79)
No. of metastatic parotid lymph nodes (3+ *vs*. 1–2)	<0.001	7.43 (3.32–16.62)
ENE	0.888	1.09 (0.35–3.39)
Deep lobe involvement	0.219	1.72 (0.72–4.09)
Neck management		
ENI		Ref
Observation	0.001	2.50 (1.45–4.30)
END	0.246	0.70 (0.38–1.28)

ENE, extranodal extension; END, elective neck dissection; ENI, elective neck irradiation.

In the Cox model for DSS, END demonstrated a similar HR of 0.62 (95%CI = 0.30–1.26) compared to ENI (*p* = 0.184). However, patients who underwent observation were associated with an additional nearly twofold risk of cancer-related mortality (HR = 2.85, 95%CI = 1.55–5.23). Immunosuppression (*p* = 0.005, HR = 5.26, 95%CI = 1.66–16.69), T3/T4 stage (*p* = 0.001, HR = 12.99, 95%CI = 2.77–60.90), and positive margin (*p* < 0.001, HR = 12.24, 95%CI = 3.31–45.25) apparently decreased DSS. Deep lobe involvement was related to a trending negative impact (*p* = 0.051, HR = 3.16, 95%CI = 0.99–10.07). Poor differentiation, PNI, or ENE was not associated with DSS (**Table 4**).

**Table 4:** Multivariate analysis of the predictors for disease-specific survival (DSS).

Variable	DSS	
	*p*-value	HR (95%CI)
Immunosuppression (yes *vs*. no)	0.005	5.26 (1.66–16.69)
Tumor stage (T3 + T4 *vs*. T1 + T2)	0.001	12.99 (2.77–60.90)
Differentiation		
Well + intermediate		Ref
Poor	0.196	2.00 (0.70–5.69)
PNI (yes *vs*. no)	0.321	2.32 (0.54–7.19)
Positive margin	<0.001	12.24 (3.31–45.25)
No. of metastatic parotid lymph nodes (3+ *vs*. 1–2)	0.001	5.74 (2.08–15.86)
ENE	0.679	1.29 (0.39–4.23)
Deep lobe involvement	0.051	3.16 (0.99–10.07)
Neck management		
ENI		Ref
Observation	0.001	2.85 (1.55–5.23)
END	0.184	0.62 (0.30–1.26)

PNI, perineural invasion; ENE, extranodal extension; END, elective neck dissection; ENI, elective neck irradiation.

### Subgroup analysis

**Figure 2:**
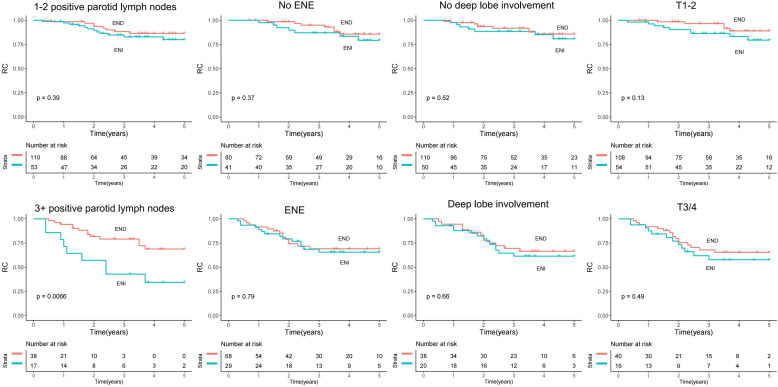
Comparison of regional control (RC) between the elective neck dissection (END) and elective neck irradiation (ENI) groups stratified by positive parotid lymph nodes (LNs), deep lobe involvement, tumor stage, and extranodal extension (ENE) of the parotid LN.

**Figure 3:**
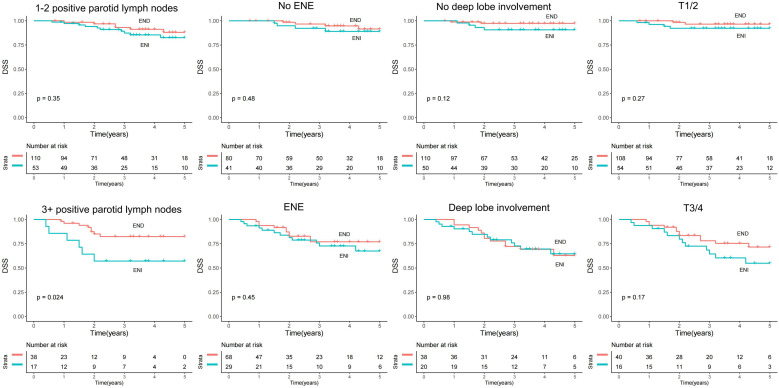
Comparison of disease-specific survival (DSS) between the elective neck dissection (END) and elective neck irradiation (ENI) groups stratified by positive parotid lymph nodes (LNs), deep lobe involvement, tumor stage, and extranodal extension (ENE) of the parotid LN.

To determine the impact of ENI and END further, their role on RC and DSS was analyzed and stratified by several important variables. In patients with one or two metastatic parotid LNs, ENI predicted comparable RC (*p* = 0.389) and DSS (*p* = 0.346) to END but offered worse RC (*p* = 0.007) and DSS (*p* = 0.024) in patients with three or more positive LNs. ENI was associated with non-inferior RC and DSS compared to END independent of tumor stage, ENE, and deep lobe involvement (all *p* > 0.05) (**Figures 2**, **3**).

### Recurrent HNcSCC treatment

Remedial surgery was successfully performed in 30 (34.9%, 30/86) patients, of whom previous management was observed in 17 (45.9%, 17/37) cases, ENI in 5 (23.8%, 5/21) cases, and END in 8 (28.6%, 8/28) cases. Patients without salvage operation were treated *via* palliative therapy, including chemotherapy, targeted drugs, and immunotherapy, among others.

## Discussion

Our most important finding was that the prognosis of P+ HNcSCC was relatively satisfactory, but observation was related to worse RC and DSS than elective neck treatment. The number rather than the ENE of parotid LNs influenced survival, and it also determined the impact of elective neck treatment. ENI provided similar RC and DSS to END in patients with one or two positive parotid LNs, but inferior survival to END in those with three or more positive LNs. Deep lobe involvement and tumor stage did not affect the role of elective neck treatment. These results offered benefits in the decision-making of neck management in P+ HNcSCC. END should always be the first option, but ENI could be an alternative method only if there were no more than two metastatic parotid LNs.

Approximate management of a cN0 neck is critical but has always been ignored in prior literature, which tended to clarify oncologic outcomes, occult cervical disease, or LN metastasis pattern in HNcSCC (13–15). To the best of our knowledge, only three studies were available for analysis of the impact of different neck managements on survival (10, 16, 17). Cannon et al. (17) might be the first to answer this question. The authors collected data from 59 cN0 HNcSCC patients: 28 cases underwent END and 31 received observation. Patients treated with END showed significantly better disease-free survival, neck control, and overall survival than those in the observation group. However, the study failed to describe the parotid LN status. Amit et al. (10) were the second to solve this issue. The authors included 1,111 patients with no evidence of nodal metastasis, of whom 173 cases were treated with END and the rest were observed. The regression model described that, compared with observation, END did not offer improved DSS or disease-free survival, but was even associated with inferior overall survival. The same situation was also observed in patients with T3/T4 disease. The finding was easily perceived. On the one hand, patients who underwent END might have had more adverse pathological features than those observed; on the other hand, salvage surgery was more commonly performed in the observation group, which could translate into an increased prognosis. However, their outcome conflicted with ours: we confirmed that observation was related to poorer RC and DSS than elective neck treatment. This difference could be explained by at least three aspects. Firstly, different populations were enrolled, and parotid metastasis in HNcSCC predicted occult cervical disease with a rate exceeding 20% (18). This was a general principle of the END requirement in head and neck squamous cell carcinoma (19). Secondly, more salvage operations were conducted in the observation group, but it did not prolong the DSS time. Delayed detection of nodal metastasis usually shows poor prognosis, and this was quite confirmed in head and neck cancer (20). Thirdly, the recurrence pattern in the observation group was more complex, which was associated with a higher possibility of positive margin occurrence.

The third study aimed to clarify whether ENI could replace END without survival compromise (16). A total of 107 patients with P+ HNcSCC were analyzed. Of these, 42 patients received END followed by radiotherapy, while 65 patients were treated with ENI alone. There was only one cervical recurrence in each group, and the difference was not significant. The authors concluded that ENI with a dose of approximately 50–60 Gy could be an alternative method for END. The finding was largely consistent with ours: ENI tended to have a smaller protective role than END, as reflected by the HR, but it was not significant. To determine the indication for ENI and END further, a subgroup analysis was performed. The number of metastatic parotid LNs served as a key factor, and the presence of three or more positive LNs determined that ENI was not suitable in P+ HNcSCC. This finding was interesting: firstly, parotid LN was likely to be a sentinel for cervical LN. A high parotid metastasis burden usually meant more chance and a greater number of positive neck LNs (21), and ENI alone was always not good at controlling an N2/N3 neck. Secondly, END provided an accurate neck stage. Patients with no occult metastasis might be exempted from radiation, and this offered a chance of a second operation, but it was usually not possible after ENI.

ENE was an indicator of poor prognosis and aggressive treatment, but its role in HNcSCC has been rarely analyzed. The presence of ENE was found to be less frequent in the observation group than in the END and ENI groups (*p* = 0.046). This finding was understandable as the decision to avoid END might have been guided by the presence of minimal nodal involvement in the parotid region, making the sparing of END a reasonable and appropriate option. However, we failed to describe an association between ENE and RC or DSS. The finding might be equal to that in parotid cancer: the parotid LN usually has a smaller size than the cervical LN, and its capsule is thinner than that of the cervical LN. ENE even presented in 41.0% of our sample, but it might be reflected by anatomical rather than biological features (12, 22). Subgroup analysis revealed that ENE did not determine the impact of END and ENI. This finding was quite important. Personal experience predominated during the clinical decision-making, and a suggestion of END was highly given if there was the presence of ENE. Our results might alter this arbitrary situation, and ENI did not decrease the prognosis.

One or two LNs were located within the deep lobe of the parotid gland. They had a rich lymphatic network with cervical LN (23) and usually acted as an adverse prognostic indicator and predicted a higher possibility of neck nodal disease and tumor recurrence in parotid cancer (24). However, we noted that deep lobe involvement was not related to RC and exhibited a trending negative impact on DSS. The effect of END or ENI was not influenced by deep lobe status. A possible explanation was that deep lobe metastasis was relatively uncommon, only occurring in 25.4% of our sample, and the majority underwent a total parotidectomy.

T3/T4 stage was a high-risk factor in HNcSCC and linked with an increased rate of occult nodal metastasis. Prior knowledge proved that END rather than observation was required in the presence of a T3/T4 stage (16). Our study also supported this viewpoint partly because it was shown that ENI could achieve comparable survival to END in advanced HNcSCC, as previously described by Herman et al. (17). Immunosuppression was not only linked to an increased incidence of HNcSCC development, but it was also associated with a worse prognosis. Tam et al. (25) found that, in their study, which included 796 patients, the 5-year DSS was 68.2% in the immunosuppression group compared to 84.1% in the non-immunosuppression group. The difference was statistically significant, and multivariate analysis revealed that immunosuppression provided an additional 1.5-fold risk of death caused by cancer. A recent meta-analysis (26) confirmed that immunosuppression led to a worse prognosis across all outcome variables, including locoregional recurrence (HR = 2.20, 95%CI = 1.45–3.36), disease-free survival (HR = 2.69, 95%CI = 1.60–4.51), DSS (HR = 3.61, 95%CI = 2.63–4.95), and overall survival (HR = 2.09, 95%CI = 1.64–2.67). Our results supported these findings.

Limitations in the current study must be acknowledged. Firstly, there was a potential selection bias in the retrospective study as patients treated with observation tended to have a lower tumor burden. To address this issue and provide more conclusive evidence, a randomized controlled study is needed to clarify the question. Secondly, we did not compare the quality of life between the END and ENI groups. Thirdly, external validation is required before clinical application.

In summary, in P+ HNcSCC, END should always be the first option, but ENI could be an alternative method only if there were no more than two metastatic parotid LNs.

## Data availability statement

The original contributions presented in the study are included in the article/**Supplementary Material**. Further inquiries can be directed to the corresponding author.

## Ethics statement

The studies involving humans were approved by The First Affiliated Hospital of Zhengzhou University. The studies were conducted in accordance with the local legislation and institutional requirements. The participants provided their written informed consent to participate in this study.

## Author contributions

QS: Conceptualization, Data curation, Formal analysis, Investigation, Methodology, Validation, Visualization, Writing – original draft, Writing – review & editing. NX: Conceptualization, Data curation, Formal analysis, Investigation, Methodology, Project administration, Resources, Software, Supervision, Validation, Visualization, Writing – original draft, Writing – review & editing.

## Supplementary material

Supplementary Figure 1 Survival plots of patients with different clinicopathologic features.
