# Supplementary figures and images for: Neck management in metastatic cutaneous squamous cell carcinoma of the head and neck
Source: Front Oncol. 2024 Feb 29;14:1344115. doi: 10.3389/fonc.2024.1344115 (PMC10937539; doi:10.3389/fonc.2024.1344115)

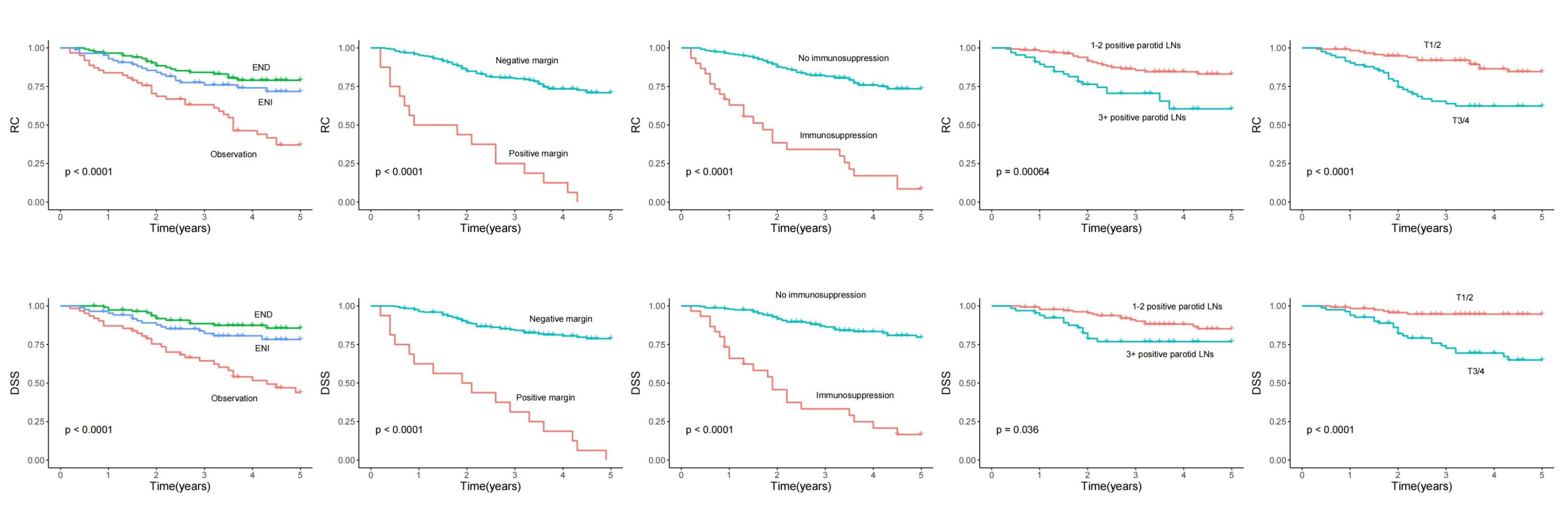

Supplement: Supplementary Figure 1 — Survival plots of patients with different clinicopathologic features. [file Image_1.jpeg]
